# Draw-Induced Structural Optimization of PAN-Based Carbon Fibers During High-Temperature Carbonization

**DOI:** 10.3390/nano15171335

**Published:** 2025-08-30

**Authors:** Seungmin Yu, Hyun-Jae Cho, Tae-Hoon Ko, Hak-Yong Kim, Yong-Sik Chung, Byoung-Suhk Kim

**Affiliations:** 1 Department of JBNU-KIST Industry-Academia Convergence Research, Jeonbuk National University, 567 Baekjedaero, Deokjin-gu, Jeonju-si 54896, Jeollabuk-do, Republic of Korea; 2 Department of Organic Materials and Textile Engineering, Jeonbuk National University, 567 Baekjedaero, Deokjin-gu, Jeonju-si 54896, Jeollabuk-do, Republic of Korea; 3 Department of Nano Convergence Engineering, Jeonbuk National University, 567 Baekjedaero, Deokjin-gu, Jeonju-si 54896, Jeollabuk-do, Republic of Korea

**Keywords:** PAN-based carbon, tensile strain, microstructure, high modulus

## Abstract

This study investigates the effect of tensile strain during high-temperature carbonization on the microstructural development and mechanical properties of polyacrylonitrile (PAN)-based carbon fibers. The wet-spun stabilized PAN precursor fibers were carbonized at 1400 °C under various tensile draw ratios (0%, 5%, 10%, and 15%), followed by stress-free graphitization at 2400 °C in an argon atmosphere for 1 h to isolate the effects of the carbonization-stage tension. Structural characterization using XRD, 2D-XRD, Raman spectroscopy, and HR-TEM revealed that moderate tensile strain (5–10%) promoted significant improvements in crystallinity, orientation, and graphene layer alignment. Notably, the fiber drawn at 10% performed the best, with a reduced interlayer spacing (*d_002_*), increased lateral crystallite size (*L_a_*), high orientation factor, and minimal turbostratic disorder. These structural developments translated into the best mechanical properties, including a tensile strength of ~2.44 GPa, a Young’s modulus of ~408.6 GPa, and the highest measured density (1.831 g/cm^3^). In contrast, excessive strain (15%) induced microstructural defects and reduced performance, underscoring the detrimental effects of overstretching. The findings highlight the critical role of draw control during carbonization in optimizing the structure–property relationships of carbon fibers, offering valuable insight for the design of high-performance fiber processing strategies.

## 1. Introduction

High-performance carbon fibers (HPCFs) are extensively utilized in aerospace structures, high-end automotive components, sporting goods, wind turbine blades, and next-generation energy storage devices, where weight reduction and mechanical reliability are of paramount importance [1]. As global demand for lightweight yet durable structural materials continues to rise, the development of carbon fibers with optimized processing and superior performance remains a critical research focus. Among various precursors, polyacrylonitrile (PAN)-based carbon fibers dominate the market owing to their superior balance of processability and final mechanical performance [2]. The properties of PAN-based carbon fibers are strongly governed by microstructural evolution during the stabilization and carbonization steps, particularly in terms of crystallite growth, orientation, and defect formation [3,4]. Therefore, understanding and controlling the processing parameters that govern this microstructural development is essential for achieving further improvements in the performance of HPCFs. Such HPCFs derive their mechanical properties from an oriented, graphitic microstructure composed of stacked graphene layers aligned along the fiber axis [5,6,7]. Achieving a high degree of graphitic order typically requires heat treatment at very high temperature (2000–3000 °C), known as graphitization. This increases both the carbon content and crystallite size, thereby enhancing the Young’s modulus of the fibers. For example, transforming PAN-based carbon fibers from an intermediate modulus (~200 GPa) to a high modulus (>300 GPa) generally requires a final heat treatment at or above 2500 °C [8]. However, heat treatment without applied tension often leads to fiber shrinkage and the formation of misaligned or turbostratic structures, which limits the achievable modulus. It is well established that applying tensile stress during high-temperature treatment (commonly referred to as “stress graphitization”) promotes the alignment of graphene layers along the fiber axis, thereby improving both modulus and electrical conductivity [9]. Li et al. [10] investigated the effect of multiple stretching during high-temperature graphitization of PAN-based carbon fibers and found that repeated 2% strain at 2200 °C enhanced crystal alignment and improved the tensile modulus without compromising strength. Qian et al. [11] developed PAN-based ultrahigh modulus carbon fibers by applying high tension during the graphitization of high-strength precursor fibers, demonstrating that the use of well-aligned, high-strength intermediates is critical for achieving exceptional mechanical properties. Indeed, mechanical stresses applied during the critical stage of heat treatment can significantly enhance molecular alignment and facilitate graphitization of carbon precursors. For instance, stretching certain precursor fibers (e.g., rayon-based) by ~100% during graphitization can produce nearly perfectly aligned graphene layers, yielding ultra-high-modulus fibers (>500 GPa). However, implementing such extreme high-temperature stretching is technologically challenging and costly. Moreover, PAN-based carbon fibers, which are stronger than rayon-based carbon fibers, cannot sustain very large strains at extreme temperatures without damage. In most commercial PAN-based carbon fiber production systems, some degree of tension is applied during both the stabilization and carbonization steps to prevent fiber shrinkage and avoid slack. Meanwhile, the role of stretching during the carbonization step (typically conducted at 1000–1500 °C) has long been a subject of debate. While slight tension is commonly applied to maintain fiber alignment, excessive strain at this stage may introduce defects, and the optimal strain level for improving properties without inducing damage remains unclear [12]. Previous studies have predominantly focused on the effects of tension during the final graphitization or stabilization stages [13,14,15]. However, there is a gap in our understanding of whether moderate, continuous tensile strain during the carbonization step at mid-range temperatures might precondition the fiber’s microstructure for subsequent graphitization. In other words, can a fiber that is partially oriented at 1400 °C later graphitize more efficiently at 2400 °C, even without concurrent tension? Addressing this question is important because maintaining tension at ~2500 °C requires specialized equipment and incurs high costs, whereas applying tension at a lower temperature (when the fiber is stronger and equipment is more readily available) could be a more practical approach if it yields comparable benefits.

In this work, we systematically investigate the effect of tensile drawing during high-temperature carbonization on the microstructures and properties of PAN-based fibers. The wet-spun, stabilized PAN precursor fibers were initially carbonized at a low-temperature of 800 °C, and followed by continuous high-temperature carbonization at 1400 °C under controlled tension (draw ratios ranging from 0% to 15%). This was followed by graphitization at 2400 °C without applying tension. In PAN-based carbon fiber processing, the high-temperature carbonization region (1150–1800 °C) is primarily associated with the gasification of residual heteroatoms such as nitrogen and oxygen, rather than with graphitic crystallite growth. This gasification process can generate microvoids and micropores within the fiber interior. Applying tensile stress during this stage can suppress the formation of such defects and simultaneously promote molecular alignment, thereby improving the final structural order and mechanical properties. This rationale supports our choice of 1400 °C as the representative condition for applying controlled tensile strain [16,17]. Applying tension stress at 1400 °C is therefore expected to facilitate chain alignment and enhance graphitic ordering, owing to the reduced internal resistance associated with this transitional microstructure state [18]. The evolution of fiber structure was characterized using Raman spectroscopy and wide-angle X-ray diffraction (including 2D diffraction for orientation analysis), and these findings were correlated with the changes in density and mechanical properties (tensile modulus and strength). A draw ratio of about 10% during carbonization was found to optimize the microstructure, yielding a carbonized fiber with significantly reduced Raman *I*_D_/*I*_G_ value, decreased XRD interlayer spacing (*d*_002_), and improved orientation compared to undrawn fibers. After graphitization, the graphitized fiber (10GF) that had undergone 10% drawing retained these structural advantages, achieving the highest degree of graphitic order and the highest modulus (~408.6 GPa) with only a slight reduction in tensile strength. In contrast, the graphitized fiber (0GF) carbonized without tension showed inferior alignment and limited strength and modulus, while the graphitized fiber (15GF) subjected to excessive drawing (15%) exhibited microstructural degradation that offset the benefits of improved orientation. These results highlight that a moderate pre-strain during carbonization is an effective way to enhance crystallite development and mechanical performance in PAN-based carbon fibers, offering a practical strategy to produce high-modulus fibers without the need for tension during the ultra-high temperature stage.

## 2. Experimental

### 2.1. Materials

The acrylonitrile (AN, ≥99%) and itaconic acid (IA, ≥99%) used for the synthesis of PAN precursors were purchased from Samchun Chemicals (Pyeongtaek, Republic of Korea). Dimethyl sulfoxide (DMSO, 99.9%) and 2,2′-azobisisobutyronitrile (AIBN, ≥99.0%) were obtained from Daejung Chemicals & Metals (Siheung, Republic of Korea). The 1-Dodecanethiol (DDM, ≥98.0%) used as a chain transfer agent was purchased from Daejung Chemical & Metals (Siheung, Republic of Korea). All chemicals were used as received without additional purification. The PAN polymer was synthesized via conventional free-radical polymerization, following procedures described in the literature [19]. The average molecular weight of the obtained polymer was approximately 103 kDa (by GPC), and the solution viscosity was 49.2 Pa·s.

### 2.2. Preparation of Carbon and Graphitized Fibers

**Scheme 1:**
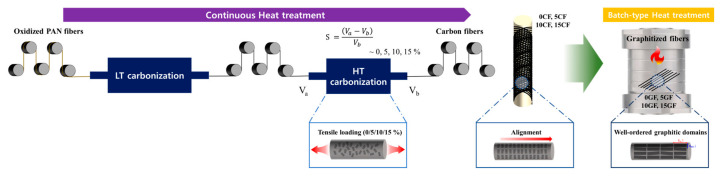
Schematic diagram of low-temperature (LT) and high-temperature (HT) carbonizations, and subsequent bath-type graphitization. Various tensile strains are applied in the stage of high-temperature (HT) carbonization zone.

A spinning dope (~21 wt%) was prepared by dissolving the synthesized PAN polymer in DMSO. The PAN precursor fibers were fabricated using a custom-designed wet spinning system [20]. As-spun PAN precursor fibers were subjected to oxidative stabilization in air using multi-zone stabilization furnaces (DISSOL Co., Ltd., Gimje, Republic of Korea), with the temperature progressively increasing from 190 °C to 260 °C over a period of 90 min. The stabilized PAN fibers were then continuously processed through a low-temperature carbonization (LT) furnace operating at 400–800 °C, followed by a high-temperature carbonization (HT) furnace at 1400 °C under a nitrogen atmosphere. The residence times in the LT and HT stages were approximately 5.0 min and 1.75 min, respectively. During high-temperature carbonization, stretching was applied by controlling the take-up and feed roller speeds at both ends of the HT furnace. The stretching ratio (*S*) applied during this process was defined by the following Equation (1):

(1) $$S=(V_{a}-V_{b})/V_{b}$$

where *V_a_* is the fiber speed at the exit of the HT furnace and *V_b_* is the speed at the entry. The applied tensile strains were set to 0%, 5%, 10%, and 15%, and the resulting carbon fibers were designated as 0CF, 5CF, 10CF, and 15CF, respectively. Subsequently, each carbonized sample was graphitized in a batch-type furnace at 2400 °C for 1 h under an argon atmosphere. The resultant graphitized fibers were denoted as 0GF, 5GF, 10GF, and 15GF, respectively. The procedure is schematically represented in Scheme 1.

### 2.3. Characterization

X-ray diffraction (XRD) was performed using a diffractometer (Rigaku, Tokyo, Japan) with Cu Kα radiation to evaluate the crystalline structure. The interlayer spacing (*d_hkl_*) was calculated using Bragg’s law (2) [21]:

(2) $$d_{hkl}=\frac{n\lambda }{2sin\theta_{hkl}}$$

where *λ* is the wavelength of the X-ray source (1.5406 Å) and *θ_hkl_* is the diffracted angle, respectively.

The crystallite stacking height (*L_c_*) and crystallite lateral size (*L_a_*) were estimated using the Scherrer Equations (3) and (4) [22]:

(3) $$L_{c}=\frac{0.89\lambda }{\beta_{002}cos\theta_{002}}$$

(4) $$L_{a}=\frac{1.84\lambda }{\beta_{100}cos\theta_{100}}$$

where *λ* is the wavelength of the X-ray source, *β_hkl_* is the full-width at half maximum (FWHM), and *θ_hkl_* is the diffracted angle.

Raman spectroscopy (LabRam GR UV/Vis/NIR, Horiba, Kyoto, Japan) with a 514 nm excitation laser was used to evaluate the graphitic structure. The spectra were deconvoluted into multiple bands: the D band (~1350 cm^−1^, defect-induced breathing mode of sp^2^ rings), the D_2_ band (~1620 cm^−1^, a shoulder of the G band associated with edge defects or stacking disorder), the D_3_ band (~1500 cm^−1^, attributed to amorphous carbon), the D_4_ band (~1200 cm^−1^, related to C–C stretching in disordered regions), the G band (~1580 cm^−1^, E_2g_ vibration mode of sp^2^ carbon), and the 2D band (~2700 cm^−1^, a second-order two-phonon process reflecting graphitic stacking order) [23]. The *I*_D_/*I*_G_ values were obtained from the integrated peak areas after multi-peak fitting, serving as indicators of defect content, while the *I*_2D_/*I*_G_ ratios were determined from the peak intensities, reflecting the degree of graphene layer stacking. Furthermore, the in-plane crystallite size (*L*_*a*,Raman_) was estimated using the Tuinstra–Koenig relation (5) [24]:

(5) $$L_{a,Raman}=\frac{2.4\times 10^{-10}\lambda^{4}}{I_{D}/I_{G}}$$

To evaluate the crystallite orientation along the fiber axis, a 2D wide-angle X-ray diffraction (2D-XRD) system equipped with a VÅNTEC-500 area detector (Bruker, Karlsruhe, Germany) was used. Azimuthal scans were performed along the circular Debye–Scherrer ring of the (002) reflection, and the diffraction intensity was recorded as a function of azimuthal angle *φ*. In this configuration, *φ* = 0° corresponds to the fiber axis. The higher intensity at *φ* = 0° indicates that the graphitic layers ((002) planes) are preferentially aligned parallel to the fiber axis, consistent with the characteristic anisotropic orientation of carbon fibers. The degree of orientation was quantitatively evaluated by calculating the Hermann orientation factor, *f_H_*, which ranges from 0 (random orientation) to 1 (perfect alignment). It is defined by then following Equation (6) [25]:

(6) $$f_{H}=\frac{3\left\langle {cos}^{2}\varphi \right\rangle -1}{2}$$

where ⟨cos^2^*φ*⟩ is the average of cos^2^*φ*, obtained from the intensity distribution of the azimuthal scan.

Fiber surface morphology was observed by scanning electron microscopy (SEM) (SU3900, HITACHI, Tokyo, Japan). Fibers were mounted on sample stubs and imaged at moderate magnification under an accelerating voltage of 10 kV. High-resolution transmission electron microscopy (HR-TEM) was used to examine the internal graphitic nanostructure of the fibers after graphitization. Thin longitudinal sections of individual fibers were prepared using focused ion beam (FIB) milling, and the specimens were observed in a TEM (Tecnai G2 F20, FEI, Hillsboro, OR, USA) operated at 80 kV. The HR-TEM images enabled direct visualization of stacking and alignment of the graphitic layer along the fiber axis. Single-fiber tensile tests were performed to evaluate the mechanical properties. Testing was conducted using a single-fiber tensile tester (FAVIMAT+, Textechno, Mönchengladbach, Germany) at a gauge length of 20 mm. For each condition, 20 individual fibers were tested to obtain average tensile strength and Young’s modulus values. For each condition, 20 individual fibers were tested, and the mean values were presented with error bars representing the standard deviations. Fiber density was measured using a density gradient column prepared with calibrated mixtures of benzene (density~0.876 g/cm^3^) and tetrabromomethane (density~2.97 g/cm^3^). The equilibrium flotation positions of the fiber samples within the column were used to determine their respective densities.

## 3. Results and Discussion

**Figure 1:**
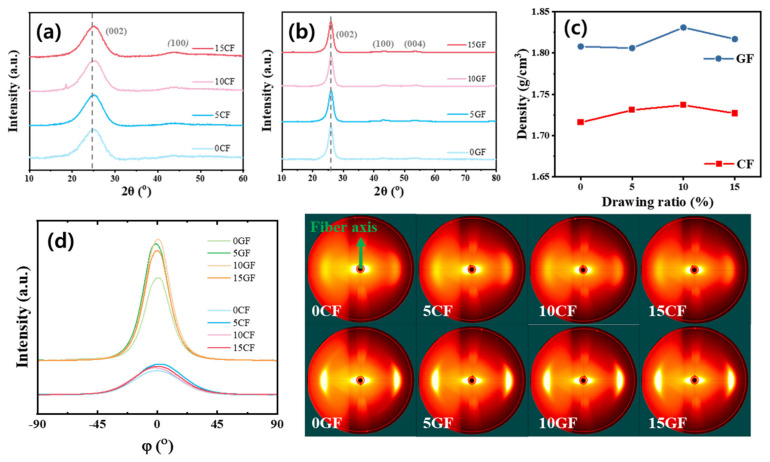
XRD patterns of (**a**) CFs after carbonization at 1400 °C, (**b**) GFs after subsequent graphitization at 2400 °C, (**c**) bulk density of CFs and GFs at various drawing ratios, Azimuthal *φ*-scan profiles of (**d**) carbon fibers (0CF, 5CF, 10CF and 15CF) and graphitized fibers (0GF, 5GF, 10GF and 15GF) with 2D XRD patterns.

The degree of tensile strain applied during the high-temperature carbonization of PAN-based precursor fibers was found to significantly influence the microstructure and mechanical properties of the resulting carbon fibers. According to X-ray diffraction (XRD) and two-dimensional wide-angle X-ray diffraction (2D-XRD) analyses, applying an appropriate tensile stress during continuous carbonization at approximately 1400 °C induced alignment of the graphitic layers along the fiber axis and enhanced crystallinity. As shown in Figure 1a, the (002) diffraction peak of the carbon fiber without tension (0CF) was slightly shifted to a lower angle, indicating a relatively large interlayer spacing (*d_002_*) of approximately 3.61 Å and a broader peak profile. In contrast, the fiber subjected to 10% tension (10CF) exhibited a decreased d-spacing of ~3.58 Å, suggesting a reduced interlayer distance and a structure closer to crystalline carbon [26]. Furthermore, as shown in Figure 1d, the azimuthal intensity distribution (*φ*-scan) narrowed with increasing tension, reflecting an improved degree of orientation of the crystallites along the fiber axis. As a result, the bulk density increased from approximately 1.72 g/cm^3^ (0% tension) to ~1.74 g/cm^3^ at 10% tension (Figure 1c). This increase in density is a natural consequence of crystal growth and densification, and it is well known that the carbon content and density of carbon fibers tend to rise with increasing crystallinity during high-temperature treatment [27]. In the case of excessive tension (15%), the carbon fiber (15CF) did not exhibit further reduction in d-spacing, and in some instances, the *d_002_* value slightly increased (≈3.583 Å), indicating structural saturation or signs of abnormal microstructural distortion. These results suggest that overstraining can disturb microstructural development, whereas a moderate draw ratio between 5 and 10% is most effective in promoting the alignment and crystallization of the graphitic structure. The corresponding values are summarized in Table 1.

**Figure 2:**
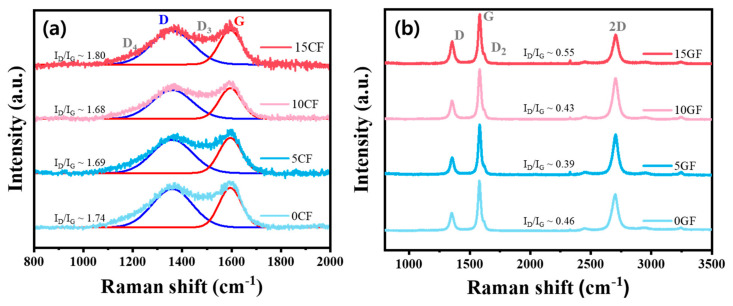
Raman spectra of (**a**) CFs before and (**b**) GFs after graphitization.

Raman spectroscopic analysis (Figure 2) revealed trends consistent with the structural changes observed in XRD. In the carbonized fibers, the integrated intensity ratio of the D band to the G band (*I*_D_/*I*_G_) for the sample without applied tension (0CF) was approximately 1.74, indicating a high amount of amorphous and defect-rich carbon. However, in the fiber subjected to 10% tensile strain, the *I*_D_/*I*_G_ ratio significantly decreased to around 1.68, suggesting a marked reduction in structural disorder. This implies that the tensile strain facilitated more ordered alignment of graphene layers, thereby reducing the number of structural imperfections [28]. In contrast, the *I*_D_/*I*_G_ ratio increased again to approximately 1.80 under 15% strain, indicating a substantial rise in disorder. This degradation is attributed to overstretching-induced microcracks or structural damage, which leads to an increased presence of defect-related carbon species. After the subsequent graphitization at 2400 °C (Figure 2b), the *I*_D_/*I*_G_ ratio for all samples was significantly reduced, indicating that high-temperature treatment promoted overall graphitization [29]. Notably, fibers drawn at 5–10% maintained very low D/G ratios (≤0.43) even after graphitization, reflecting a high content of well-ordered graphitic carbon. In contrast, the 15% drawn fiber retained a higher D/G ratio exceeding 0.5, implying that more defects remained compared to other conditions. These results suggest that tensile strain up to approximately 10% is beneficial for promoting the growth of graphitic domains, while excessive strain induces permanent structural imperfections that are not fully healed during graphitization. Moreover, the 2D band of the graphitized fibers also exhibited clear tension-dependent changes. The *I*_2D_/*I*_G_ ratio reached a maximum of ~0.82 at 10% tensile strain, which was substantially higher than that of the undrawn sample (~0.73). Since the intensity of the 2D band is closely related to the stacking order of graphene layers, the prominent 2D peak under 10% tension indicates that the graphitic structure achieved optimal stacking and alignment [30]. On the other hand, the weakened 2D band in the 15% drawn fiber suggests disruption of this ideal layered arrangement due to overstretching. Furthermore, the in-plane crystallite size (*L_a_*_,Raman_) was evaluated from Raman spectra using the Tuinstra–Koenig relation. The calculated values, summarized in Table 2, exhibit a consistent trend with the XRD-derived *L_a_* values, demonstrating that moderate tensile strain (5–10%) facilitates crystallite growth, whereas excessive strain (15%) leads to defect accumulation and reduced *L_a_*_,Raman_.

**Figure 3:**
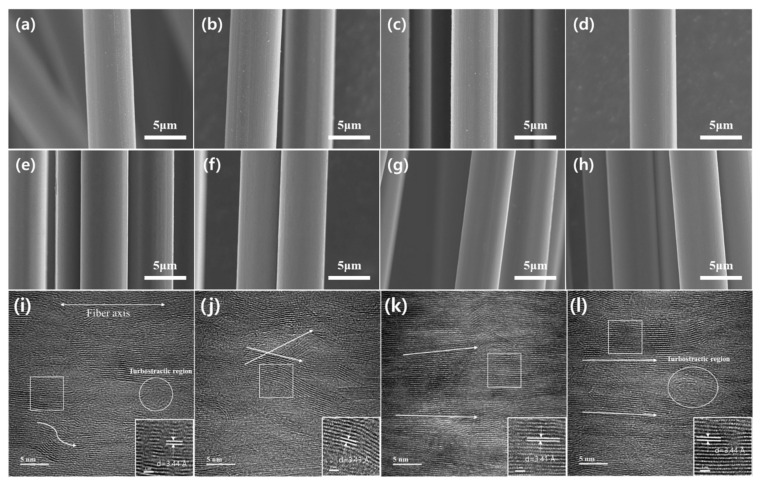
SEM images of carbon fibers (CFs, (**a**) 0CF, (**b**) 5CF, (**c**) 10CF, (**d**) 15CF) and graphitized fibers (GFs, (**e**) 0GF, (**f**) 5GF, (**g**) 10GF, (**h**) 15GF), HR-TEM images of graphitized fibers (GFs, (**i**) 0GF, (**j**) 5GF, (**k**) 10GF, (**l**) 15GF). The rectangular boxes are shown as magnified TEM images of the corresponding samples in inset. The circles show the turbostratic region. The arrows indicate the direction of graphitic alignment.

Microscopic observations using SEM and TEM clearly revealed the structural differences induced by tensile strain. As shown in the SEM images of carbonized fibers (Figure 3a–d), fibers subjected to tensile stress during high-temperature carbonization exhibited slightly elongated and oriented surface textures along the fiber axis. In contrast, the surfaces of the graphitized fibers (Figure 3e–h) showed minimal differences across all samples, suggesting that the subsequent high-temperature graphitization process smoothed out surface irregularities, resulting in uniformly smooth fiber surfaces. High-resolution TEM images (Figure 3i–l) further revealed notable differences in internal microstructure depending on the applied tensile strain. The graphitized fiber without tension (0GF) displayed a significant number of turbostratic regions, characterized by the disordered stacking of carbon layers. In comparison, the 10% drawn sample (10GF) exhibited a well-aligned internal structure, where graphene layers were predominantly arranged parallel to the fiber axis, forming long and continuous graphitic domains. Although the 15% drawn sample also showed an overall tendency for axial alignment, local regions marked by circles in the TEM images revealed signs of misalignment, interlayer twisting, and voids indicative of residual turbostratic structure [31].

**Figure 4:**
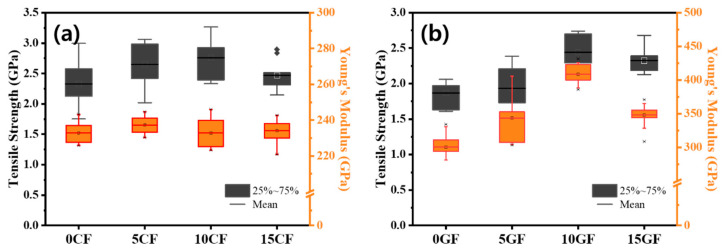
Tensile strength (gray) and Young’s modulus (orange) of (**a**) carbon fibers (CFs; 0CF, 5CF, 10CF, and 15CF) and (**b**) graphitized fibers (GFs; 0GF, 5GF, 10GF, and 15GF) under different strain conditions.

These observations suggest that a moderate level of tensile strain facilitates the alignment of graphitic layers by pulling the carbon sheets taut along the fiber axis. However, excessive tension may disrupt this alignment, leading to partial layer detachment or slippage, and ultimately resulting in locally defective structures. These structural changes were directly reflected in the mechanical performance of the carbon fibers. The tensile strength and modulus of individual fibers, measured through single-fiber tensile testing (Figure 4), revealed clear trends depending on the applied draw ratio. For carbonized fibers at 1400 °C, the tensile strength increased from approximately 2.3 GPa for the undrawn sample (0CF) to 2.6 GPa at 5% draw and reached a maximum of 2.76 GPa at 10% draw. However, at 15% draw, the strength dropped to 2.47 GPa, indicating a threshold beyond which excessive tension compromises fiber strength. In contrast, the tensile modulus showed only minor changes in the carbonized state (ranging from ~233 to 237 GPa), but a dramatic increase was observed after graphitization. The modulus of the undrawn graphitized fiber (0GF) was approximately 300 GPa, whereas fibers drawn at 5% and 10% exhibited substantially higher values of 349 GPa and 408 GPa, respectively. This enhancement can be attributed to the formation of highly aligned and well-crystallized structures induced by the applied tension. On the other hand, the fiber drawn at 15% exhibited a reduced modulus of ~344 GPa, significantly lower than that of the 10% sample. As previously discussed, this decrease is likely due to accumulated structural damage and limited crystal growth caused by overstretching, which hinder the fiber’s ability to efficiently transfer applied stress [32,33]. The performance degradation at 15% strain can be attributed to excessive tensile stress exceeding the structural tolerance of turbostratic carbon layers. Such overstretching is likely to induce interlayer slippage, partial delamination, or microcrack formation within the graphitic domains, thereby introducing irreversible defects that cannot be fully healed during subsequent graphitization. This interpretation is supported by structural evidence: the (002) interlayer spacing slightly increased, and the bulk density decreased at 15% strain, both of which indicate a higher degree of turbostratic disorder and the formation of voids within the fiber structure. Consequently, the fiber drawn at 10% demonstrated the best mechanical performance after graphitization, with a tensile strength of approximately 2.4 GPa and a modulus of ~409 GPa. In contrast, both properties were diminished in the 15% drawn fiber, confirming that excessive tensile strain adversely affects the mechanical properties of carbon fibers. An analysis of the correlations among various structural and mechanical parameters revealed a strong interdependence between the development of crystallinity and the enhancement of mechanical performance.

**Figure 5:**
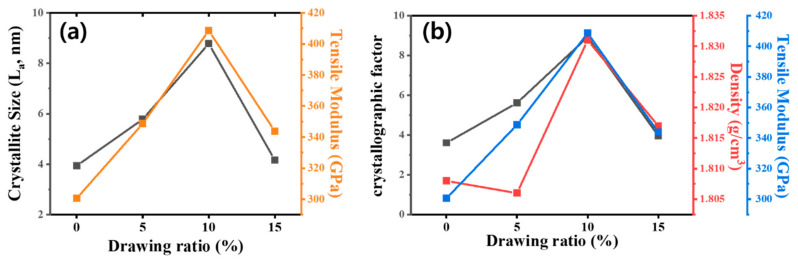
(**a**) Relationship between in-plane crystallite size (*L_a_*) and tensile modulus of graphitized fibers (GFs), (**b**) correlation of tensile modulus with bulk density and a crystallographic factor defined as (*L_a_* × *L_c_*/*d_002_* × *f_H_* × *I*_2D_/*I*_G_).

As illustrated in Figure 5a, a clear positive correlation was observed between the lateral crystallite size (*L_a_*) and the tensile modulus in the graphitized fibers. For example, the undrawn sample (0GF) exhibited an *L_a_* of approximately 3.9 nm and a tensile modulus of ~300 GPa, whereas the 10% drawn fiber showed a significantly increased *L_a_* of 8.8 nm and a modulus exceeding 400 GPa. This trend indicates that as the graphene layers grow larger and become more extended along the fiber axis, with increasing *L_a_*, the stiffness of the fiber correspondingly improves [34]. In addition, the enhanced axial orientation induced by tension contributed notably to the increased modulus. The graphitized fiber drawn at 10% exhibited an orientation factor (*f_H_*), measured by XRD, of approximately 0.905, which is only slightly higher than that of the undrawn fiber (0.903). However, it is likely that the pre-existing alignment developed during the carbonization stage played a key role in amplifying the final modulus after graphitization.

To further quantify the structure–property relationship, we propose a crystallographic factor as a composite structural index that combines multiple descriptors of graphitic order. Specifically, we define an index as *L_a_* × (*L_c_*/*d_002_*) × *f_H_* × (*I*_2D_/*I*_G_) for the graphitized fibers, incorporating the in-plane crystallite size, out-of-plane crystallite size, interlayer spacing, orientation factor, and Raman 2D-band intensity ratio, respectively. As shown in Figure 5b, this composite factor correlates positively with the Young’s modulus across the graphitized samples. In particular, the 10GF fiber with the highest modulus also exhibits the highest value of this index, whereas the over-drawn 15GF shows a slightly lower index consistent with its reduced modulus due to its larger *d_002_* and lower *I*_2D_/*I*_G_. This suggests that the proposed structural index effectively captures the contributions of crystallite size, alignment, and lattice order to the fiber’s stiffness. It is noteworthy that this correlation is applicable only to highly graphitic fibers that display a well-defined 2D Raman band (such as the graphitized samples in this study); turbostratic carbon fibers without a 2D band would yield a near-zero *I*_2D_/*I*_G_, making the index meaningless. Nevertheless, for the fully graphitized high-modulus fibers examined here, the index provides a concise quantitative link between microstructural perfection and tensile modulus.

In summary, the concurrent increase in crystallinity, orientation, and density formed an interconnected microstructural framework that enhanced both the tensile modulus and strength of the carbon fibers. These results underscore the importance of applying an optimal level of tensile strain (around 10% in this work) to maximize the mechanical properties through structural refinement. The underlying reason for the optimal mechanical performance observed near a 10% draw ratio can be clearly understood through the microstructural and spectroscopic analyses presented above. During the carbonization process, applying approximately 10% tensile strain effectively counteracts the natural shrinkage of the fibers, allowing the graphene layers to interlock and align along the fiber axis. As a result, a more continuous and defect-free graphitic structure is formed, which facilitates substantial crystallite growth (i.e., increased *L_a_*) and the development of a well-organized three-dimensional network during the subsequent graphitization at 2400 °C. Under these optimal conditions, microvoids and incomplete bonding sites that could act as defects are minimized, enabling the retention of both high tensile strength and high modulus after graphitization [35]. In contrast, when no tension is applied, the fibers undergo uncontrolled shrinkage during carbonization, causing the graphene layers to fold and twist in a disordered current. This leads to a discontinuous structure with small, misaligned crystallites. Although such fibers may show relatively high orientation values in 2D-XRD measurements, their true crystalline quality is low, with abundant structural defects, ultimately limiting their mechanical performance. As a result, fibers processed without tension exhibited inferior modulus and strength, along with restricted crystallization during graphitization. On the other hand, applying excessive tensile stress during high-temperature carbonization may exceed the strain tolerance of the fiber, leading to local cracking or interlayer slippage. That is, some graphene layers may rupture or delaminate before achieving full alignment, resulting in microvoids and incomplete stacking [36]. Such damage hinders crystallite development, as evidenced by the reduced *L_a_* and increased Raman D/G ratio in the 15% drawn fibers after graphitization, and compromises the uniformity of the fiber bundle by potentially breaking weaker filaments or inducing cross-sectional thinning. Consequently, both tensile strength and modulus declined under the 15% draw conditions. These findings demonstrate that overstretching accumulates structural defects and irregular deformation within the fibers, ultimately negating the benefits of crystallization and orientation and degrading the mechanical properties. In conclusion, controlling the draw ratio during the high-temperature carbonization of PAN-based carbon fibers offers a powerful means to tailor the final structural and mechanical properties. A moderate tensile strain (approximately 5–10%) promotes the alignment and growth of graphitic layers, resulting in reduced interlayer spacing, enlarged crystallites, enhanced orientation, and increased density. These microstructural improvements translate into simultaneous gains in tensile strength and modulus. In particular, a 10% draw condition yielded the highest degree of structural order and continuous graphene alignment along the fiber axis, thereby optimizing mechanical performance. In contrast, excessive strain (≥15%) increased structural instability and defect formation, which disrupted crystallization and alignment, decreasing the mechanical properties. These results highlight the critical importance of precisely controlling tensile strain during carbonization to produce high-performance carbon fibers and underscore the role of draw optimization in balancing graphitic structure development with property enhancement.

## 4. Conclusions

In this study, PAN-based precursor fibers were subjected to high-temperature carbonization at 1400 °C under varying draw ratios (0%, 5%, 10%, and 15%), and subsequent structural and mechanical changes in both the carbonized and graphitized fibers were investigated to isolate the effect of tension at the carbonization stage. Graphitization was performed in a batch-type furnace at 2400 °C to ensure consistent post-treatment conditions. XRD and Raman analyses revealed that under moderate tension (5–10%), the (002) interlayer spacing of the graphitized fibers decreased, crystallite sizes increased, the integrated intensity of D/G ratio significantly decreased, and the 2D band intensity increased, indicating enhanced crystallinity and graphene layer alignment. Correspondingly, the density, tensile strength, and Young’s modulus of the fibers improved markedly, with the 10% drawn fiber (10GF) exhibiting the best overall properties, including the highest density (1.831 g/cm^3^), a Young’s modulus of approximately 408.6 GPa, and a tensile strength of ~2.44 GPa. These improvements were further supported by 2D-XRD *φ*-scan profiles and HR-TEM observations, which confirmed optimal microstructural alignment along the fiber axis and a minimal presence of turbostratic disorder in the 10% drawn sample. In contrast, excessive tension (15%) introduced microcracks and defects due to internal stress accumulation during carbonization, which led to increased structural disorder—evidenced by a higher Raman D/G ratio and smaller crystallite size—and ultimately degraded the mechanical performance. These results demonstrate that appropriate tensile control during the carbonization process—approximately 10% draw in this study—can effectively enhance both the microstructure and properties of carbon fibers. From a scientific perspective, this work quantitatively elucidates the crystallization behavior of carbon fibers under varying tensile conditions, contributing to a deeper understanding of carbon structure development. From an industrial viewpoint, it provides a viable processing strategy to maximize structural development prior to graphitization, enabling the production of high-strength, high-modulus carbon fibers through optimal draw control during carbonization.

## Figures and Tables

**Table 1:** Structural parameters and densities of carbon fibers (CFs) and graphitized fibers (GFs) treated with various drawing ratios.

Samples	*d_002_* (Å)	*d_100_* (Å)	*L_c_*	*L_a_*	*f_H_*	Density (g/cm^3^)
0CF	3.609	2.072	1.741	4.704	0.728	1.716
5CF	3.589	2.073	1.711	3.566	0.778	1.731
10CF	3.582	2.075	1.686	4.394	0.768	1.737
15CF	3.583	2.081	1.727	4.261	0.773	1.727
0GF	3.439	2.068	4.767	3.942	0.903	1.808
5GF	3.435	2.069	4.681	5.787	0.879	1.806
10GF	3.432	2.080	4.701	8.782	0.905	1.831
15GF	3.439	2.069	4.792	5.128	0.909	1.817

**Table 2:** Raman-derived structural parameters of CFs: intensity ratios (*I*_D_/*I*_G_, *I*_2D_/*I*_G_) and in-plane crystallite size (*L_a_*) calculated using the Tuinstra–Koenig relation (λ = 514 nm).

Samples	*I*_D_/*I*_G_	*I*_2D_/*I*_G_	*L_a,_*_Raman_ (nm)
0CF	1.74	-	2.53
5CF	1.69	-	2.60
10CF	1.68	-	2.62
15CF	1.80	-	2.44
0GF	0.46	0.73	9.57
5GF	0.39	0.81	11.28
10GF	0.43	0.82	10.23
15GF	0.55	0.61	8.00

## Data Availability

Data are contained within the article.

## References

[1] Tanaka F. (2024). Pioneering the carbon fiber frontier: A half-century of industry leadership and the road ahead. Compos. Part B Eng..

[2] Malik H., He J., Zhang H., Liu Y., Yu J. (2025). Computational insights into PAN-based carbon fiber advancements: Simulation techniques, microstructural-property relationship, and future directions. Int. Mater. Rev..

[3] Zhu H., Chen Q., Malik H., Wang Y., He J., Ma B. (2024). A comparison of the effect of oxygen partial pressure on microstructural evolution of PAN fibers during industrial carbon fiber production line at different altitudes. Polym. Degrad. Stab..

[4] Choi J., Jeon C., Lee J.E., Lee G.H., Hwang S., Han M. (2025). Optimization of the carbonization process based on the evolution of microstructural components of polyacrylonitrile (PAN)-based fibers. Carbon.

[5] Li D., Lu C., Wang L., Du S., Yang Y. (2017). A reconsideration of the relationship between structural features and mechanical properties of carbon fibers. Mater. Sci. Eng. A.

[6] Jang D., Lee M.E., Choi J., Cho S.Y., Lee S. (2022). Strategies for the production of PAN-based carbon fibers with high tensile strength. Carbon.

[7] Lee J.-E., Choi J., Lee D.J., Lee S., Chae H.G. (2022). Radial microstructure development of polyacrylonitrile (PAN)-based carbon fibers. Carbon.

[8] Jiang Z., Chen Q., Fan B., He J., Malik H., Zhang H., Liu Y., Yu J. (2025). Microstructure and properties of PAN-based carbon fiber with different graphitization temperature (up to 3000 °C). Diam. Relat. Mater..

[9] Ye C., Wu H., Huang D., Li B., Shen K., Yang J., Liu J., Li X. (2019). The microstructures and mechanical properties of ultra-high-strength PAN-based carbon fibers during graphitization under a constant stretching. Carbon Lett..

[10] Li S., Cui D., Wang Y., Gao A., Tong Y. (2025). Effect of multiple stretching on microstructure and mechanical properties of PAN-based carbon fibers. Carbon Lett..

[11] Qian X., Zhi J., Chen L., Zhong J., Wang X., Zhang Y., Song S. (2018). Evolution of microstructure and electrical property in the conversion of high strength carbon fiber to high modulus and ultrahigh modulus carbon fiber. Compos. Part A Appl. Sci. Manuf..

[12] Liu R., Wu H., Ye G., Shi K., Huang D., Quan H., Ye C., Zhu S., Fan Z., Qian F. (2025). Positive effect of isotropic components on the elongation at break of mesophase pitch-based carbon fibers. ACS Omega.

[13] Soulis S., Konstantopoulos G., Koumoulos E.P., Charitidis C.A. (2020). Impact of alternative stabilization strategies for the production of PAN-based carbon fibers with high performance. Fibers.

[14] Qin X., Lu Y., Xiao H., Zhao W. (2013). Effect of heating and stretching polyacrylonitrile precursor fibers in steam on the properties of stabilized fibers and carbon fibers. Polym. Eng. Sci..

[15] Li D., Wang H., Wang X. (2007). Effect of microstructure on the modulus of PAN-based carbon fibers during high temperature treatment and hot stretching graphitization. J. Mater. Sci..

[16] Morgan P. (2005). Carbon Fibers and Their Composites.

[17] Park M., Jang D., Endo M., Lee S., Lee D.S. (2022). The effect of heat treatment on temperature-dependent transport and magnetoresistance in polyacrylonitrile-based carbon fibers. Mater. Des..

[18] Dhakate S.R., Bahl O.P. (2003). Effect of carbon fiber surface functional groups on the mechanical properties of carbon–carbon composites with HTT. Carbon.

[19] Kim T., Kim B.-S., Ko T.H., Kim H.Y. (2025). Effect of boron and iron at various concentrations on the catalytic graphitization of the polyacrylonitrile derived from the polymerization of acrylonitrile. Inorganics.

[20] Cho H.-J., Lee H.R., Kim B.-S., Chung Y.-S. (2024). Catalytic effects on graphitized carbon fibers of graphitization catalysts introduced during hot-water stretching. Compos. Res..

[21] Pope C.G. (1997). X-ray diffraction and the Bragg equation. J. Chem. Educ..

[22] Holzwarth U., Gibson N. (2011). The Scherrer equation versus the ‘Debye-Scherrer equation’. Nat. Nanotechnol..

[23] Mishakov I.V., Stepanov A.G., Prosvirin I.P., Cherepanova S.V., Vedyagin A.A., Shubin Y.V., Kuznetsov V.L. (2022). Water purification from chlorobenzenes using heteroatom-functionalized carbon nanofibers produced on self-organizing Ni–Pd catalyst. J. Environ. Chem. Eng..

[24] Tuinstra F., Koenig J.L. (1970). Raman spectrum of graphite. J. Chem. Phys..

[25] Joshi K., Arefev M.I., Zhigilei L.V. (2019). Generation and characterization of carbon fiber microstructures by atomistic simulations. Carbon.

[26] Aladekomo J.B., Bragg R.H. (1990). Structural transformations induced in graphite by grinding: Analysis of 002 X-ray diffraction line profiles. Carbon.

[27] Cermak M., Perez N., Collins M., Bahrami M. (2020). Material properties and structure of natural graphite sheet. Sci. Rep..

[28] Ferrari A.C., Robertson J. (2000). Interpretation of Raman spectra of disordered and amorphous carbon. Phys. Rev. B.

[29] Liu F., Wang H., Xue L., Fan L., Zhu Z. (2008). Effect of microstructure on the mechanical properties of PAN-based carbon fibers during high-temperature graphitization. J. Mater. Sci..

[30] Popov V.N., Lambin P. (2013). Theoretical 2D Raman band of strained graphene. Phys. Rev. B.

[31] Musiol P., Szatkowski P., Gubernat M., Weselucha-Birczynska A., Blazewicz S. (2016). Comparative study of the structure and microstructure of PAN-based nano- and micro-carbon fibers. Ceram. Int..

[32] Wang M.-l., Bian W.-f. (2020). The relationship between the mechanical properties and microstructures of carbon fibers. New Carbon Mater..

[33] Ruan R.-y., Ye L.-w., Feng H., Xu L.-H., Wang Y. (2020). High temperature evolution of the microstructure in the radial direction of PAN-based carbon fibers and its relationship to mechanical properties. New Carbon Mater..

[34] Qin X., Lu Y., Xiao H., Wen Y., Yu T. (2012). A comparison of the effect of graphitization on microstructures and properties of polyacrylonitrile and mesophase pitch-based carbon fibers. Carbon.

[35] Naito K., Tanaka Y., Yang J.-M., Kagawa Y. (2008). Tensile properties of ultrahigh strength PAN-based, ultrahigh modulus pitch-based and high ductility pitch-based carbon fibers. Carbon.

[36] Ozbek S., Isaac D.H. (2000). Strain-induced density changes in PAN-based carbon fibres. Carbon.

